# The leptin system and its expression at different nutritional and pregnant stages in lined seahorse (*Hippocampus erectus*)

**DOI:** 10.1242/bio.020750

**Published:** 2016-09-14

**Authors:** Huixian Zhang, Geng Qin, Yanhong Zhang, Shuisheng Li, Qiang Lin

**Affiliations:** 1 CAS Key Laboratory of Tropical Marine Bio-resources and Ecology, South China Sea Institute of Oceanology, Chinese Academy of Sciences, Guangzhou, Guangdong 510301, China; 2 State Key Laboratory of Biocontrol, School of Life Sciences, Sun Yat-Sen University, Guangzhou, Guangdong 510275, China

**Keywords:** Leptin, Leptin receptor, Nutrient, Pregnancy, Seahorse

## Abstract

Leptin is an essential hormone for the regulation of energy metabolism and food intake in vertebrate animals. To better understand the physiological roles of leptin in nutrient regulation in paternal ovoviviparous fish (family Syngnathidae), the present study cloned the full-length of *leptin-a* and leptin receptor (*lepr*) genes in lined seahorse (*Hippocampus erectus*). Results showed that there was a 576-bp intron between two exons in *leptin-a* gene but no *leptin-b* gene in seahorse. Although the primary amino acid sequence conservation of seahorse *leptin-a* was very low, the 3-D structure modeling of seahorse *leptin-a* revealed strong conservation of tertiary structure with other vertebrates. Seahorse *leptin-a* mRNA was highly expressed in brain, whereas *lepr* mRNA was mainly expressed in ovary and gill. Interestingly, both *leptin-a* and *lepr* mRNA were expressed in the brood pouch of male seahorse, suggesting the leptin system plays a role during the male pregnancy. Physiological experiments showed that the expression of hepatic *leptin-a* and *lepr* mRNA in unfed seahorses was significantly higher than that in those fed 100%, as well as 60%, of their food during the fasting stage, showing that seahorse might initiate the leptin system to regulate its energy metabolism while starving. Moreover, the expression of *leptin-a* in the brood pouch of pregnant seahorse was significantly upregulated compared with non-pregnant seahorse, whereas the expression of *lepr* was downregulated, suggesting that the leptin system might be involved in the male pregnancy. In conclusion, the leptin system plays a role in the energy metabolism and food intake, and might provide new insights into molecular regulation of male pregnancy in seahorse.

## Abstract (precis)

**Summary:** This study provided insights into the function of the leptin system in energy metabolism regulation and male pregnancy in lined seahorse (*Hippocampus erectus*).

## INTRODUCTION

Seahorses, which belong to the family Syngnathidae, are ovoviviparous fish whose embryos can obtain paternal nutrients during pregnancy through the male's brood pouch and maternal nutrients from yolk (Foster and Vincent, 2004; Wilson et al., 2001). Seahorses mainly feed on planktonic crustaceans, such as copepods, amphipods, decapods and mysid shrimps (Kitsos et al., 2008; Lin et al., 2009, 2010). However, sometimes seahorses might have to endure starvation because of their slow swimming ability and the patchiness of prey distribution and abundance when they are taken to a new place by water current in the wild, which often leads to high mortality, especially during the juvenile seahorse stage (Lourie et al., 1999; Vincent et al., 2011). Nonetheless, the molecular mechanism of energy regulation during seahorse starvation stage is still unknown.

In teleosts and mammals, feeding is generally regulated by a number of peptides produced in brain and peripheral tissues, such as leptin (Schwartz et al., 2000). Leptin is an important hormone synthesized by the adipocytes which signal the peripheral energy reserves to the brain and regulate development, growth, energy metabolism and reproduction in mammals (Anubhuti and Arora, 2008). As the protein product of the *obese* (*ob*) gene, leptin is a kind of type-I cytokine hormone secreted by the adipocytes that acts upon the central nervous system to regulate food intake and energy metabolism in mammals (Morton et al., 2006). In teleosts, the existence of *leptin* was first demonstrated in the pufferfish (*Takifugu rubripes*) through the synteny analysis compared to mammal leptin (Kurokawa et al., 2005). Since then, the *leptin* genes have been identified in many fish species, such as the common carp (Huising et al., 2006), zebrafish (Gorissen et al., 2009), Japanese medaka (Kurokawa and Murashita, 2009), rainbow trout (*Oncorhynchus mykiss*) (Pfundt et al., 2009), Atlantic salmon (*Salmo salar*) (Ronnestad et al., 2010), grass carp (*Ctenopharyngodon idellus*) (Li et al., 2010), Arctic charr (*Salvelinus alpines*) (Froiland et al., 2010), orange-spotted grouper (*Epinephelus coioides*) (Zhang et al., 2013), among others. Although the primary sequence conservation of *leptin* in teleosts is extremely low, the secondary and tertiary structure of the protein is highly conserved (Denver et al., 2011). In contrast to mammals, the *leptin* of ectotherm vertebrates, including fish, is rarely expressed in adipose tissue and is instead mainly expressed in the liver, brain and gonads in fish (Copeland et al., 2011).

Leptin stimulates downstream genes by binding to a variety of receptors. Several forms of leptin receptor have been identified in mammals (Tartaglia et al., 1995) and amphibians (Crespi and Denver, 2006). One long form and five short isoforms have been reported in mammals, and only a long form of leptin receptor has the intracellular functional domains (Tartaglia, 1997). In teleosts, different isoforms of *lepr* have also been identified. Five different *lepr* isoforms have been found in Atlantic salmon (*Salmo salar*) (Ronnestad et al., 2010), and three *lepr* isoforms have been identified in crucian carp (*Carassius carassius*) (Cao et al., 2011). The long form is the only one that conserves all the functionally important domains in mammals, which include two JAK2 boxes and one STAT box (Ronnestad et al., 2010).

The family Syngnathidae is a special fish group because of its ovoviviparous reproductive pattern through the male's brood pouch. Interestingly, there is a gestation time before the offspring of seahorse are released (Wilson et al., 2001). The paternal nutrients provided to seahorse embryos during pregnancy are essential, in addition to the maternal nutrients from the yolk (Linton and Soloff, 1964). Therefore, the study of the regulation for nutrient transition and pregnancy of these special animals may help to understand the evolutionary adaptability of nutrient regulation in teleosts. The lined seahorse (*Hippocampus erectus*) is a highly valued species in both traditional Chinese medicine and aquarium trades, and it also has been widely used for some scientific research because it can be easily bred in a laboratory (Lin et al., 2012, 2008; Qin et al., 2014). Lined seahorse is mainly found along the western Atlantic coast and Gulf of Mexico from Florida to Nova Scotia (Foster and Vincent, 2004). *H. erectus* has been included on the IUCN list of Threatened Species as ‘Vulnerable’ (Lourie et al., 1999). The present study aimed to identify and characterize the leptin system in lined seahorse, and analyze the expression profiles of leptin system during nutrient transition and pregnant stages to detect the physiological roles of leptin system in male pregnancy species.

## RESULTS

### Characterization of *leptin-a* and *lepr* genes

**Fig. 1.**
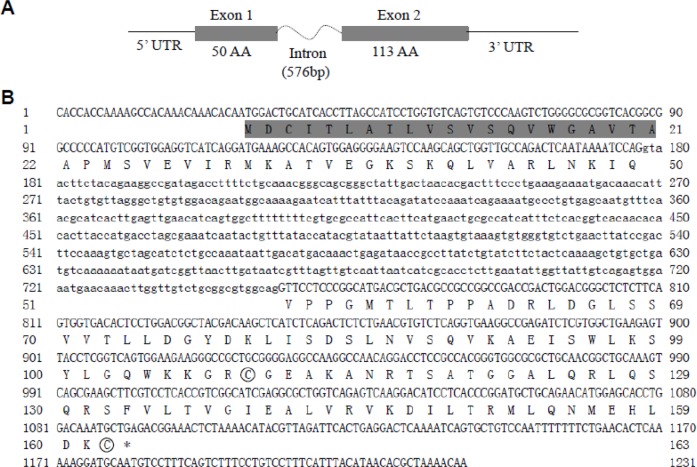
**The nucleotide and deduced protein sequences of leptin cDNA in the lined seahorse *Hippocampus erectus*.** (A) Gene structure. The boxes represent coding exons. The numbers show the base pairs and amino acids (aa). (B) The signal peptide is shown in shadow. The cysteine residues used in disulfide linkages are circled.

Full-length cDNA sequences of *leptin-a* and *lepr* genes in adult seahorse were obtained through RACE-PCR. The leptin gene was 656 bp in length and contained an open reading frame (ORF) of 489 bp (GenBank Accession No. KP888952). The deduced *leptin-a* protein was composed of 163 amino acid residues, with a 21-amino-acid signal region and a 141-amino-acid mature peptide. A 576-bp intron between the two exons was identified in seahorse (Fig. 1).

**Fig. 2.**
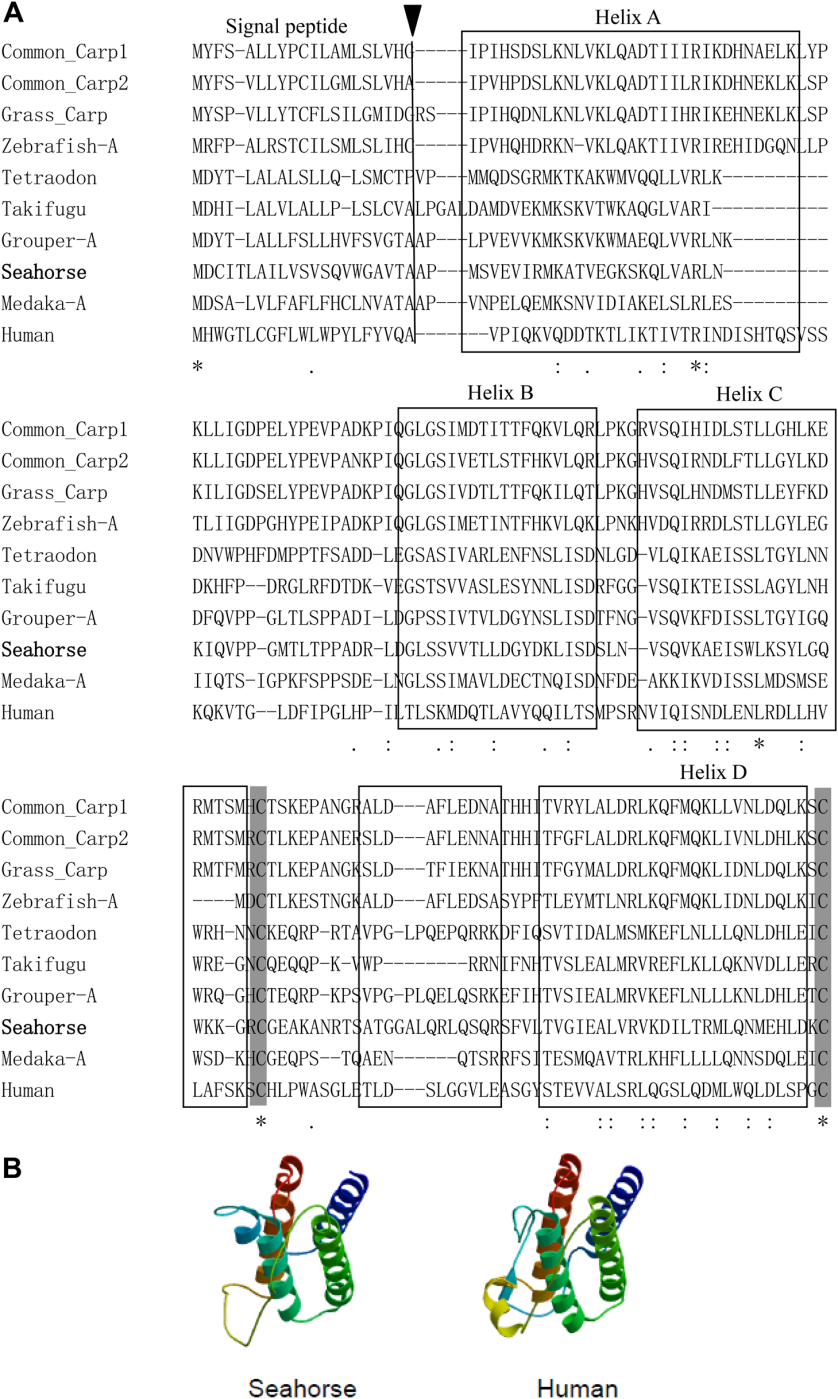
**The molecular characterization of vertebrate leptin.** (A) The comparison of amino acid sequences of the teleost and human leptins. The multiple sequence alignment was performed by ClustalX2.0. The signal peptides are indicated by a black triangle. The four α-helices of human leptin are boxed. The conserved cysteine residues involved in the formation of disulfide bridges are shaded. (B) The tertiary structures of seahorse and human leptins. The secondary and tertiary protein structures were modeled using the ProModII program at the SWISS-MODEL automated protein modeling server, based upon human leptin (1AX8.pdb) Protein Data Bank structure file.

Multiple sequence alignment was performed based on the amino acid sequences of vertebrate leptins (Fig. 2A). The deduced amino acid sequences of *leptin-a* in seahorse displayed a low identity with other vertebrate leptins, but the 3-D structure modeling showed a strong conservation of the tertiary structure of leptin between seahorse and human, as both 3-D structures had the characteristics of four-helix bundle topology and a disulfide bond (Fig. 2B). A phylogeny analysis of the mature proteins revealed that vertebrate leptin sequences clustered into two groups. The first encompassed teleost *leptin-b* sequences, and the other encompassed teleost *leptin-a* and tetrapod sequences. The seahorse *leptin-a* sequence branched within the Acanthopterygian *leptin-a* clade (Fig. S2A).

The seahorse *lepr* cDNA (GenBank Accession No. KP888953) contained a 3351-bp ORF with a coding potential for a 1116-amino acid (aa) protein, with one 22-aa signal peptide region, one 795-aa extracellular segment, one 23-aa single transmembrane domain and one 298-aa intracellular segment (Fig. S1). The *lepr* in seahorse had all functionally important domains which are conserved among vertebrate leptin receptors. All of these leptin receptors included two JAK2-binding motif boxes and one STAT-binding domain at the intracellular segment. On the basis of the phylogeny analysis of leptin receptor proteins, the *lepr* in seahorse can be grouped in a teleost branch and clustered with orange-spotted grouper (Fig. S2B).

### Tissue distribution of *leptin-a* and *lepr* genes

**Fig. 3.**
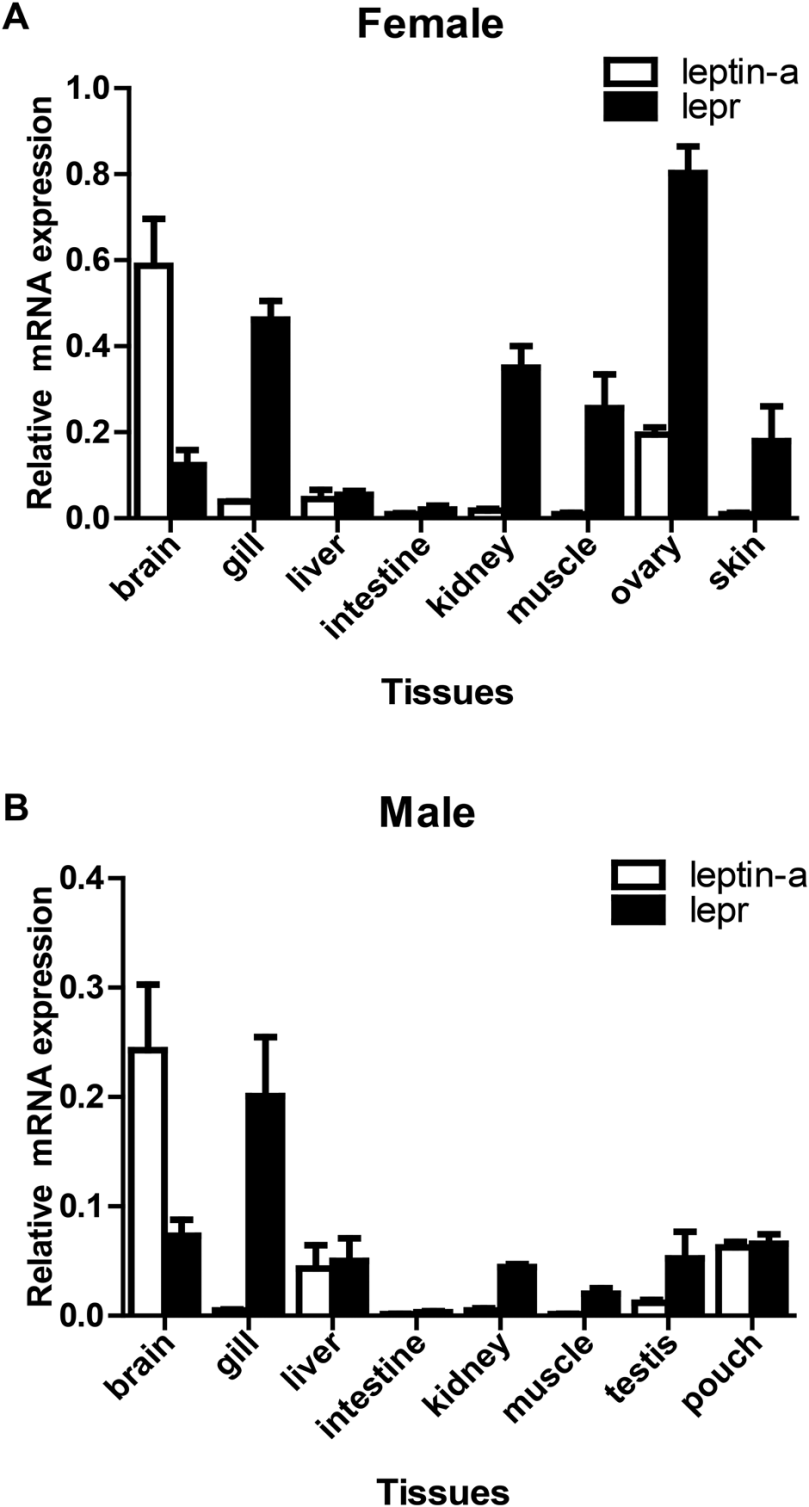
**The tissue expression of *leptin-a* and *lepr* mRNA in seahorses (*Hippocampus erectus*).** (A) Female and (B) male seahorses *Hippocampus erectus* (*n*=3). mRNA levels identified by RT-PCR normalized against β-actin transcript, including brain, gill, liver, intestine, kidney, muscle, testis (male), ovary (female), brood pouch (male), and skin (female) tissues.

The *leptin-a* mRNA was mainly expressed in brain, liver and brood pouch of male seahorses (Fig. 3B). In female seahorse *leptin-a* was expressed mainly in brain and ovary (Fig. 3A). In contrast, in male seahorse *lepr* was expressed in gill, brain, liver, kidney and brood pouch (Fig. 3B), and *lepr* was highly expressed in ovary, gill, kidney, muscle and skin in female seahorse (Fig. 3A). Interestingly, both *leptin-a* and *lepr* genes were expressed in brood pouch of male seahorse.

### Expression profiles of the leptin system at different nutritional and pregnant stages

**Fig. 4.**
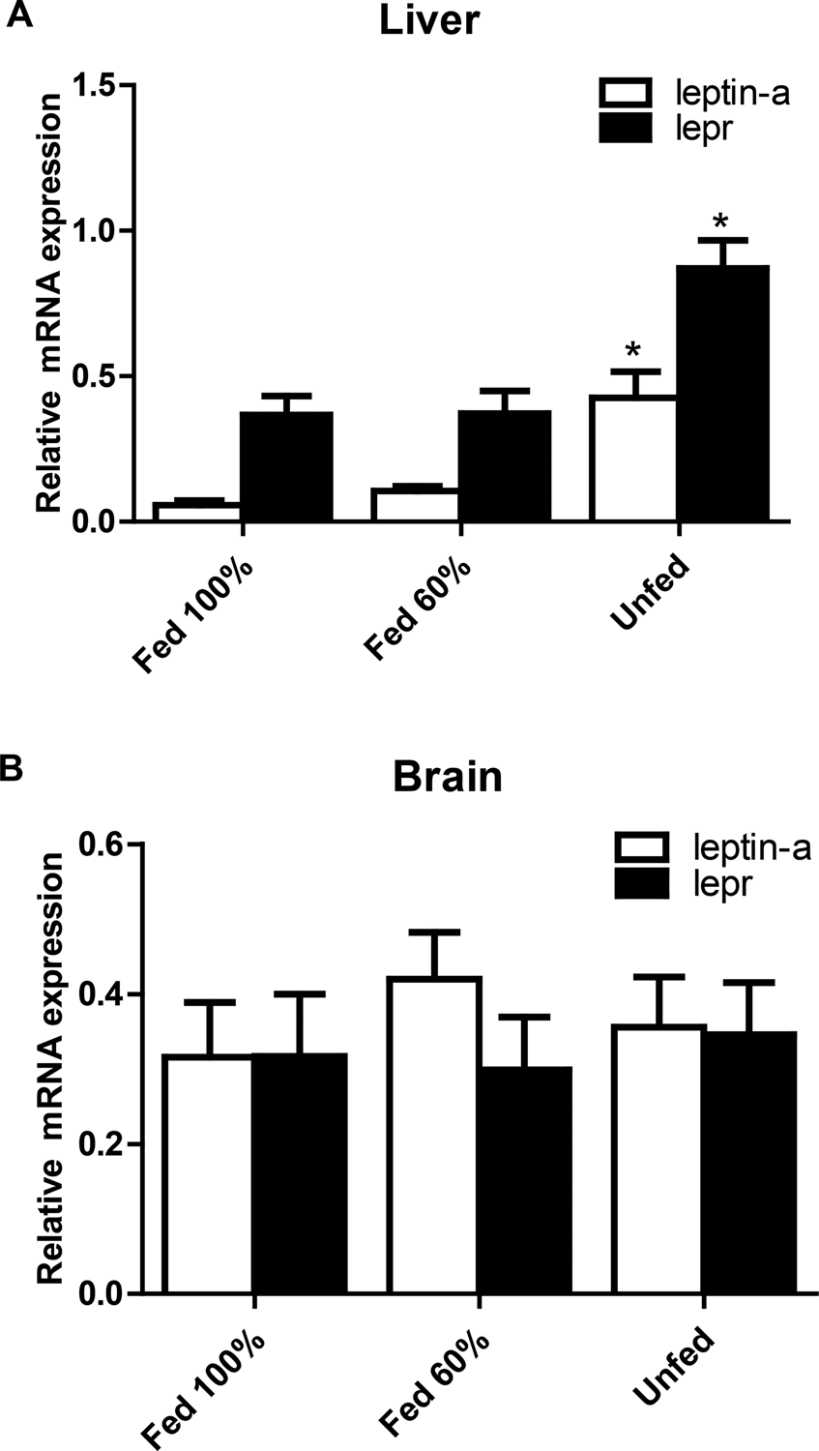
**The expression of *leptin-a* and *lepr* mRNA of juvenile seahorses (*Hippocampus erectus*) under different food intake statuses for 7 days.** (A) Liver, (B) brain (*n*=8). mRNA levels were qualified by real-time PCR. Asterisks denote significant differences between the different food intake statuses (*P*<0.05; one-way ANOVA followed by the Duncan's multiple-range tests).

The expression of hepatic *leptin-a* and *lepr* mRNA in unfed seahorses was significantly higher than those of 100% (*leptin-a*: *P*=0.014, *lepr*: *P*=0.034; *P*<0.05) and 60% fed seahorses (*leptin-a*: *P*=0.023, *lepr*: *P*=0.041; *P*<0.05), whereas the expression of *leptin-a* and *lepr* mRNA in brain from the unfed group was not significantly different from those in the 100% (*leptin-a*: *P*=0.695, *lepr*: *P*=0.798; *P*>0.05) and 60% fed (*leptin-a*: *P*=0.504, *lepr*: *P*=0.649; *P*>0.05) groups (Fig. 4).

**Fig. 5.**
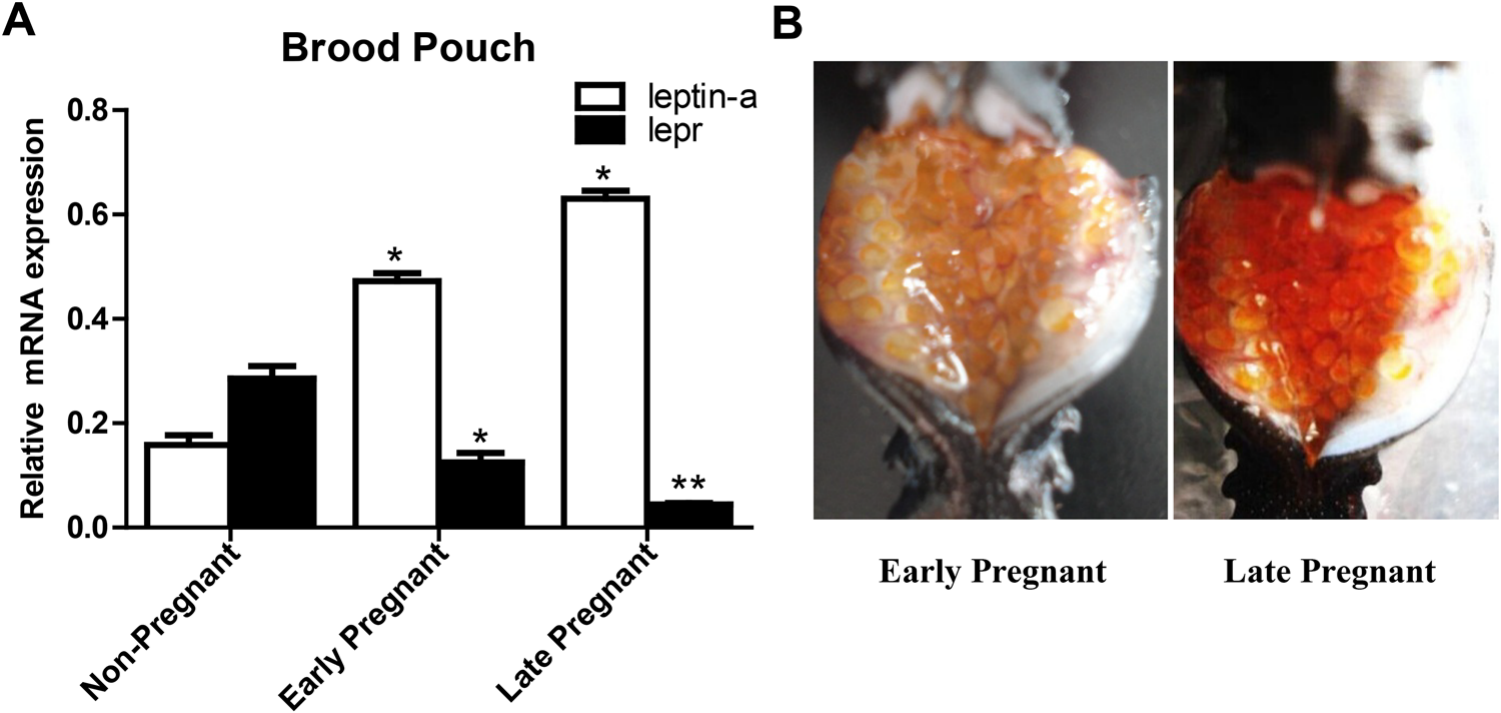
**The mRNA expressions of *leptin-a* and *lepr* during different pregnant stages.** (A)The mRNA levels were qualified by real-time PCR. The asterisks denote significant differences between pregnant and non-pregnant stages (*P*<0.05; one-way ANOVA followed by the Duncan's multiple-range tests; *n*=8). (B) The embryos attached to the brood pouch in the early pregnant stage while released to the brood pouch in the late pregnant stage.

The expression of *leptin-a* in brood pouch of early pregnant (*P*=0.173; *P*<0.05) and late pregnant (*P*=0.162; *P*<0.05) male seahorses was significantly upregulated compared with that in non-pregnant males. In contrast, the expression of *lepr* in brood pouch of early- (*P*=0.0178; *P*<0.05) and late-pregnant (*P*=0.0087; *P*<0.01) males was significantly downregulated during the pregnant stages (Fig. 5).

## DISCUSSION

The present study characterized the putative leptin system and its regulation during nutrient transition and food intake in family Syngnathidae for the first time. Fish in this family have a special nutrient compensation pattern from their parents during their unique ovoviviparous stage in male's brood pouch (Stolting and Wilson, 2007). The coding sequence of *leptin-a* in lined seahorse was most closely related to other more advanced teleosts, such as the Perciformes, Tetraodontiformes and Beloniformes (Fig. S2A). Our results showed that the amino acid sequence of *leptin-a* in seahorse shared low sequence identity with other teleosts and mammals, but its projected tertiary peptide structure showed conformational similarity to human leptin.

A 576-bp intron between two exons was found in seahorse *leptin-a*, while it is only 87 bp and 149 bp in orange-spotted grouper (Zhang et al., 2013) and Atlantic salmon (Ronnestad et al., 2010), respectively. The length of introns is positively corrected with mRNA stability and the amount of accumulated proteins (Comeron and Kreitman, 2000). The genes with longer introns produce more stable mRNAs, and finally accumulate more proteins. This suggested leptin hormone is essential to the physiological regulation in the lined seahorse.

There is only one leptin gene in mammals, whereas several teleosts possess two leptin genes. These two genes likely result from the third whole genome duplication event that occurred specifically in teleost lineage (3R). To date, two leptin forms including *leptin-a* and *leptin-b* have been identified in several fish species such as zebrafish (Gorissen et al., 2009), Japanese medaka (Kurokawa and Murashita, 2009) and orange-spotted grouper (Zhang et al., 2013). In comparison with other fish, there was no *leptin-b* gene in seahorse through the reference of seahorse genome by using tblastn and synteny analysis (Q. Lin et al., unpublished data). This finding suggests that seahorse loses one of the duplicated copies during the evolution of leptin gene.

The 3-D structural modeling of leptin predicts a strong conservation of tertiary structure between seahorse and human, including other leptins identified with four-helix bundle topology. The two conserved cysteine residues in seahorse *leptin-a* predict the formation of a disulfide bond, which is a prerequisite for tertiary structure and bioactivity of leptin in human (Rock et al., 1996). This conserved tertiary structure of leptin shows that leptin is constrained by the structure of the receptor binding pocket, and can explain why frog leptin can activate mouse leptin receptor *in vitro* (Crespi and Denver, 2006).

The protein sequence of seahorse *lepr* showed low identity with mammalian leptin recetpor, but the protein structure was similar to that of mammals (Fig. S1). A phylogenetic analysis of *lepr* proteins clearly clustered the seahorse gene within *lepr* genes in teleosts. In mammals, the alternative splicing at the 3′ end of the gene produces at least six distinct mRNA transcripts and generates several kinds of leptin receptor protein isoforms (Zabeau et al., 2003). The long-form leptin receptor identified in the present study has all functionally important domains, such as WSXWS motifs, a pair of JAK2-binding motif boxes and a STAT-binding domain. The biological activities of long-form leptin receptor via the JAK/STAT pathway in maintaining body weight and energy homeostasis have been demonstrated in mammals (Bates et al., 2003). Through the result of 3′RACE of seahorse *lepr*, there is no other short isoform of *lepr* which included the functional domains in intracellular segment of *lepr* in lined seahorse.

Seahorse *leptin-a* mRNA was mainly expressed in brain and liver, which is consistent with the pattern in Japanese medaka (Kurokawa and Murashita, 2009), Atlantic salmon (Ronnestad et al., 2010) and orange-spotted grouper (Zhang et al., 2013). The expression of *leptin-a* and *lepr* in tissues, such as kidney, heart, eye, muscle and skin, indicates that *leptin-a* has multiple functions in addition to the regulation of energy homeostasis.

The mRNA transcripts of *leptin-a* and *lepr* have been found in many peripheral tissues that have no direct relationship with feeding, such as gills and ovaries. The high expression of *lepr* in gills was also found in marine medaka (Wong et al., 2007) and crucian carp (Cao et al., 2011). These results may be related to the leptin functioning in the endocrine regulation of environmental hypoxia. The high expression of *leptin-a* and *lepr* in seahorse ovary, as found in zebrafish (Gorissen et al., 2009) and Atlantic salmon (Ronnestad et al., 2010), suggests that *leptin-a* has some functions in reproductive process in teleosts. Interestingly, both *leptin-a* and *lepr* were expressed in brood pouch of seahorse, which suggests that the leptin system might play roles during the energy transfer from male seahorses to its offspring in brood pouch.

The mRNA expression of *leptin-a* increased significantly in liver but not in brain after fasting in seahorses, suggesting that the liver is the center of energy metabolism regulation, and this result is similar to reports in goldfish (Tinoco et al., 2012) and orange-spotted grouper (Zhang et al., 2013). A significant level of hepatic *leptin-a* expression was induced by food deprivation in juvenile seahorses, but there was no change in *leptin-a* expression in seahorses subjected to rationed feeding (60% of full ration for 7 days). These results are consistent with previous studies in rainbow trout and fine flounder, which showed elevated plasma leptin levels when fasted for 1-3 weeks (Fuentes et al., 2012; Kling et al., 2009). However, the rationed feeding (60% of full ration for 10 months) in Atlantic salmon resulted in significantly reduced growth and significantly increased hepatic *leptin-a* compared with animals in a normal feeding group (Ronnestad et al., 2010). These results demonstrate that the mRNA expression of *leptin-a* in fish may increase significantly to regulate energy metabolism, allowing the animals to survive when they encounter food shortages for an extended period of time.

When the seahorses live in food-deficient conditions, they will endocrine the leptin hormone to regulate its energy metabolism to suit the environmental condition. Therefore, the down-regulation of appetite leading to the suppression of physical behavior in seahorses may be a survival strategy which is energetically advantageous. Such anorexic behavioral responses can be mediated by increasing leptin levels in liver and brain, which may trigger the activation of catabolic pathways of lipid depletion and energy metabolism in seahorse; whereas in mammals, leptin has been shown to report total lipid stores to the central nervous system, such that changes in lipid stores can be sensed rapidly and physiologically adjusted to allow survival from starvation events (Ahima et al., 2000).

Previous studies suggest that leptin can regulate food intake and energy metabolism in teleost and mammals (Copeland et al., 2011; Spiegelman and Flier, 2001). Recent studies on the link between leptin and stress hormones have focused on the relationship between stress and energy metabolism. In the common carp, chronic hypoxia and food restriction elicited gradual and parallel increases in the expression of liver *leptin-a-I*, *leptin-a-II* and *lepr* (Bernier et al., 2012). Meanwhile, the plasma cortisol level in catfish was elevated after fasting (Barcellos et al., 2010), and hypoxia can trigger a significant upregulation of *leptin-a* and *lepr* in zebrafish (Chu et al., 2010). Therefore, fishes are able to survive in environments of food deprivation and oxygen stress. This implies that hormones that regulate energy metabolism such as leptin and cortisol are involved in these complex processes.

The mRNA expression of *leptin-a* in brood pouch of pregnant seahorses was significantly higher than that of non-pregnant seahorses. It has been suggested that *leptin-a* can regulate paternal energy during the pregnant stages and may function in the energy transition between the paternal body to embryos in brood pouch. In mammals, the serum levels of maternal leptin increase gradually during the first and second trimesters and become highest in late second or early third trimester (Hardie et al., 1997). These high levels are maintained throughout the remainder of gestation and decline drastically postpartum (Schubring et al., 1998). These results demonstrate the functional importance of leptin during pregnancy.

Conversely, the mRNA expression of *lepr* in brood pouch of pregnant seahorses was significantly lower than that of non-pregnant seahorses, indicating that the expression profile of leptin receptor was not synchronized with leptin in seahorse. In mammals, hyperleptinemia in maternal serum during the pregnant stage leads to central leptin resistance by downregulating OB-Rb in the hypothalamic ventromedial nuclei and increasing circulating OB-Rb (Brunton and Russell, 2008). These results demonstrate that the function of leptin at the peripheral tissues as a paracrine/autocrine factor is capable of modifying energy metabolism. In zebrafish, a leptin receptor knockout study showed that leptin played a role in the regulation of glucose homeostasis and as a gating factor in reproductive competence (Michel et al., 2016).

In conclusion, *leptin-a* and *lepr* genes were identified in lined seahorse *H. erectus*, and both *leptin-a* and *lepr* mRNA were expressed in the brood pouch of male seahorse. As the regulated hormone for seahorse nutrient transition and food intake, leptin played an essential role in regulating the physiological and behavioral responses to adapt to food deficiency. Interestingly, *leptin-a* mRNA was significantly upregulated in the brood pouch of pregnant seahorse, suggesting the functional importance of *leptin-a* during pregnancy. The present study offers new perspectives for understanding the ecological adaptability regulated by *leptin-a* in ovoviviparous family Syngnathidae.

## MATERIALS AND METHODS

### Experimental seahorses

Lined seahorses were cultured in Shenzhen Seahorse Center of the South China Sea Institute of Oceanology, Chinese Academy of Sciences (SCSIO-CAS), with animal ethics approval for experimentation granted by the Chinese Academy of Sciences. The seahorses were maintained in re-circulating holding tanks (90×70×60 cm) with seawater pumped directly from the South China sea and treated with double sand filtration. They were fed three times a day (0900, 1200, and 1600 h) with frozen *Mysis* spp. Feces, and uneaten food was siphoned off daily. Temperature, salinity, pH, light intensity, dissolved oxygen (DO), and photoperiod were maintained at (mean±s.d.) 25±0.5°C, 32±1.0‰, 7.9±0.4, 2000 lx, 6.5±0.5 mg l^−1^, and 16 h light:8 h dark, respectively. For tissue distribution analysis, three pairs of adult seahorses (body height, 15.3±1.6 cm) were collected, anesthetized with MS222 and sacrificed by decapitation. Their tissues were dissected, frozen immediately with liquid nitrogen, and stored at −80°C until RNA extraction.

### Cloning of *leptin-a* and *lepr* genes

Liver and brain tissue from adult seahorses was used to clone the *leptin-a* and *lepr* genes. Total RNA was isolated from the frozen tissue samples using TRIzol reagent (Invitrogen, USA). One microgram of isolated RNA was used to synthesize first-strand cDNA using the Genome Erase cDNA Synthesis Kit (TAKARA, Japan). The *leptin-a* and *lepr* fragment cDNA were identified in big-belly seahorse transcriptomes (Whittington et al., 2015). To amplify these cDNA fragments, specific PCR primers were designed by using Primer 5.00 (Palo Alto, CA); these primers are shown in Table 1. The full-length cDNA sequences were obtained by the 5′- and 3′-rapid amplification of cDNA ends (RACE) using BD SMART RACE cDNA Amplification Kit (Clontech, USA) (Table 1).

**Table 1.**
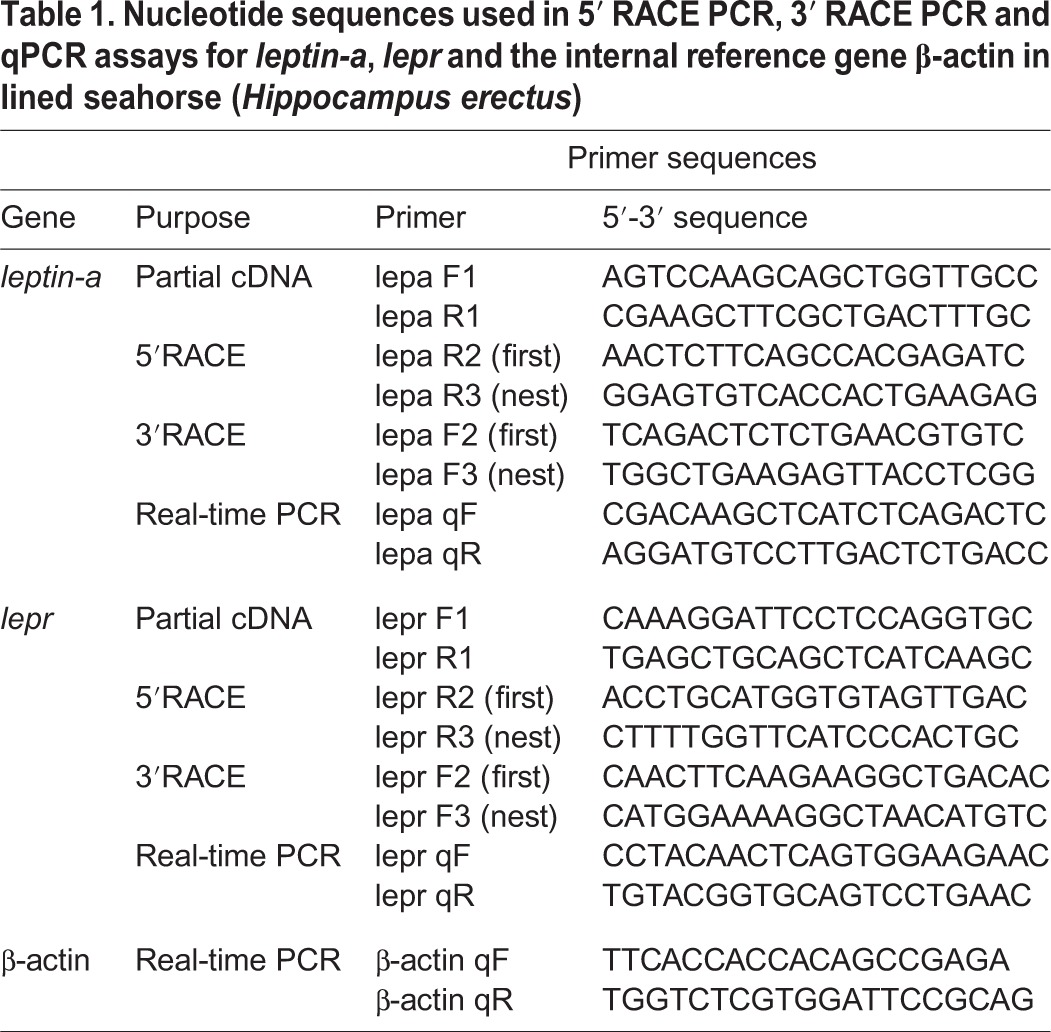
**Nucleotide sequences used in 5′ RACE PCR, 3′ RACE PCR and qPCR assays for *leptin-a*, *lepr* and the internal reference gene β-actin in lined seahorse (*Hippocampus erectus*)**

All PCR reactions in the present study were carried out using the following PCR cycling parameters: denaturation at 94°C for 3 min, followed by 35 cycles at 94°C for 20 s, 52-56°C for 20 s and 72°C for 1.5-2 min. The reaction was terminated after an extension step of 10 min at 72°C. The amplification products were purified using the E.Z.N.A Gel Extraction Kit (Omega BioTek, USA) and subcloned into the PMD18/T vector (TAKARA, Japan). Two selected clones from each amplicon were sequenced using an ABI 3700 sequencer (Applied Biosystems).

### Structural and phylogenic analysis of *leptin-a* and *lepr* genes

The *leptin-a* amino acid sequence was translated from the nucleotide sequence using DNASTAR software (Burland, 1999). The peptide structural features and tertiary configuration of mature seahorse *leptin-a* were predicted using the SWISS-MODEL automated protein modeling server (http://www.expasy.org/swissmod/SWISS-MODEL.html) (Schwede et al., 2003) based on the human leptin (1AX8A.pdb) Protein Data Bank (PDB) (http://www.rcsb.org/pdb/home/home.do). The putative signal peptides and cleavage sites of seahorse *lepr* were predicted using SignalP 3.0 (http://www.cbs.dtu.dk/services/SignalP/). The putative transmembrane domain was predicted by using TMHMM Server V2.0 (http://www.cbs.dtu.dk/services/TMHMM). Multiple sequence alignments of amino acids were performed with ClustalX2.0 (Larkin, et al., 2007). Protein phylogenetic analyses were conducted with MEGA 4.0 (Tamura et al., 2007) using the neighbor-joining method.

### Tissue expression of *leptin-a* and *lepr* mRNA

The expression patterns of *leptin-a* and *lepr* mRNA in the various tissues of the adult seahorses were analyzed by real-time PCR. Total RNA was isolated from the brains, gills, livers, intestines, kidneys, muscles, brood pouches, skin and gonads of male and female seahorses. The tissue distribution PCR primers were designed from the putative *leptin-a* and *lepr* gene-coding sequences (Table 1). The housekeeping genes β-actin and 18 s rRNA were screened by PCR in tandem on the same samples to verify the integrity of cDNA template across tissues.

### Expression profiles of *leptin-a* and *lepr* genes in juvenile seahorses at different nutritional statuses

Three treatments (100% feeding, 60% feeding and non-feeding), each with one-month seahorses (*n*=8) from the same brood, were used to compare the regulation of the *leptin-a* and *lepr* genes between fed and unfed juveniles. The seahorses in the feeding treatments were fed twice a day (0900 and 1600 h) with frozen *Mysis* spp. feces. The amount of food administered was 15% or 9% of the wet body weight of the seahorses for the 100% feeding (4.35±0.61 g; mean body weight±s.e.m.) and 60% feeding (3.87±0.52 g; mean body weight±s.e.m.) groups, respectively, while the seahorses in the non-feeding group (3.23±0.48 g; mean body weight±s.e.m.) were starved for 7 days. At 1200 h of the 8th day, six seahorses randomly collected from each tank were anesthetized by MS222, individually weighed and subsequently killed by decapitation. The liver and brain samples for quantitative RT-PCR measurement of mRNA were immediately frozen by liquid nitrogen and then stored at −80°C until RNA extraction.

### Expression profile of the leptin system during the stages of pregnancy

Adult seahorses were allowed to mate freely before being subjected to a standardized assessment of pregnancy status on the basis of courtship behaviors. Pregnant seahorses were maintained in single-sex tanks before euthanasia to sample brood pouch tissues at key stages throughout pregnancy. The targeted time periods included the following: 1-8 days post fertilization (dpf) (early pregnancy) and 9-16 dpf (late pregnancy). The embryos are attached to the brood pouch in the early pregnant stage. While in the late pregnant stage, the embryos were released from the brood pouch (Fig. 5B). We sampled six seahorses per time point (*n*=8) and detected the expression profiles of *leptin-a* and *lepr* genes by using real-time PCR.

### Quantitative real-time PCR

Then expression levels of *leptin-a* and *lepr* in lined seahorse were determined by quantitative real-time PCR (qPCR). qPCR was performed on a Roche Light-Cycler 480 real time PCR system using SYBR Premix Ex Taq™ (TAKARA, Japan) according to the manufacturer's protocol. qPCR conditions were as follows: denaturation at 94°C for 3 min, followed by 40 cycles at 94°C for 15 s, 55-58°C for 15 s and 72°C for 20 s. The standard curves of amplification for *leptin-a*, *lepr* and housekeeping genes were generated using serial dilutions of plasmid constructs as the templates. After amplification, the fluorescence data were converted to threshold cycle values (CTs). The concentration of the template in the sample was determined by relating the CT value to the standard curve (Livak and Schmittgen, 2001). The transcript levels of *leptin-a* and *lepr* were compared with the β-actin and 18 s rRNA gene transcripts.

### Statistical analyses

All the data were expressed as the means±standard error of mean (s.e.m.) and evaluated by one-way analysis of variance (ANOVA) followed by the Duncan's multiple-range tests. The results were considered to be statistically significant at a *P*-value<0.05. All statistics were using GraphPad Prism 6.0 (GraphPad Software).
